# Retraction: Long noncoding RNA ANRIL protects cardiomyocytes against hypoxia/reoxygenation injury by sponging miR-195-5p and upregulating Bcl-2

**DOI:** 10.1039/d1ra90058g

**Published:** 2021-02-03

**Authors:** Laura Fisher

**Affiliations:** Royal Society of Chemistry Thomas Graham House, Science Park, Milton Road Cambridge CB4 0WF UK advances-rsc@rsc.org

## Abstract

Retraction of ‘Long noncoding RNA ANRIL protects cardiomyocytes against hypoxia/reoxygenation injury by sponging miR-195-5p and upregulating Bcl-2’ by Hui Zhao *et al.*, *RSC Adv.*, 2019, **9**, 35624–35635, DOI: 10.1039/C9RA04898G.

The Royal Society of Chemistry hereby wholly retracts this *RSC Advances* article due to concerns with the reliability of the data. The images in the article, and raw data provided by the authors, were screened by an image integrity expert. The raw data could not be used to validate the published data as in many of the western blot panels, including Fig. 2J (Bax), Fig. 4J (Bcl-2), Fig. 5B (Bcl-2), Fig. 5B (GAPDH), Fig. 6I (Bcl-2) and Fig. 6I (Bax), the blots matched the raw data but the backgrounds did not. Furthermore, the raw data provided by the authors was found to closely resemble raw data for a number of other articles, which is unexpected given that there are completely different authors lists for these articles.

Given the significance of the concerns about the validity of both the data in the article and the raw data provided by the authors, the findings presented in this paper are not reliable.

Deguang Feng opposes the retraction. The other authors were contacted but have not responded to any correspondence regarding the retraction.

 

Signed: Laura Fisher, Executive Editor, *RSC Advances*

Date: 19^th^ January 2021

## Supplementary Material

